# Bilateral Sleeve Fracture of the Inferior Poles of the Patella in a Healthy Child: Case Report and Review of the Literature

**DOI:** 10.4061/2011/428614

**Published:** 2011-02-08

**Authors:** Stephen Paul Guy, Jan Luigi Marciniak, Nirmal Tulwa, Andrew Cohen

**Affiliations:** Pinderfields General Hospital, Aberford Road, Wakefield WF1 4DG, UK

## Abstract

The initial diagnosis of a sleeve fracture of the patella is key to a successful outcome with poor results well documented in the literature from delayed management. Diagnosis is difficult due to the rarity of this injury and thus the low likelihood the admitting junior doctor would think of this injury in their differential. They are very uncommon in incidence and have features on plain radiography that are difficult to interpret unless the surgeon is familiar with the anatomy of the immature patella. Missing the diagnosis can be disastrous for the patient. In this paper we describe the presentation of bilateral sleeve fractures in a healthy child, our initial investigations and subsequent management. We chose to repair with 5 Ethibond via 3 transosseous tunnels, initially reinforced with a circlage wire. On last review the boy maintains stable, pain-free knees with a full range of motion. The authors hope that this case and literature review will provide a valuable teaching aid and so assist in early, accurate diagnosis and cover the management options to achieve a positive outcome.

## 1. Introduction

Bilateral sleeve fractures of the patella are rare. This is the second example in English literature of this occurring in a healthy child. Often the radiological findings are overlooked due to the cartilaginous injury being far larger than the fleck of bone avulsed. An unfortunate and frequent problem encountered with sleeve fractures is the timing of the diagnosis. Delay can result in suboptimal management and outcome. We have written up this paper primarily as an interesting case report and literature review but principally to draw attention to the difficulties of diagnosis and treatment of this condition. 

The patient and his parents have given consent for the data and images surrounding the case to be published.

## 2. Case History

**Figure 1:**
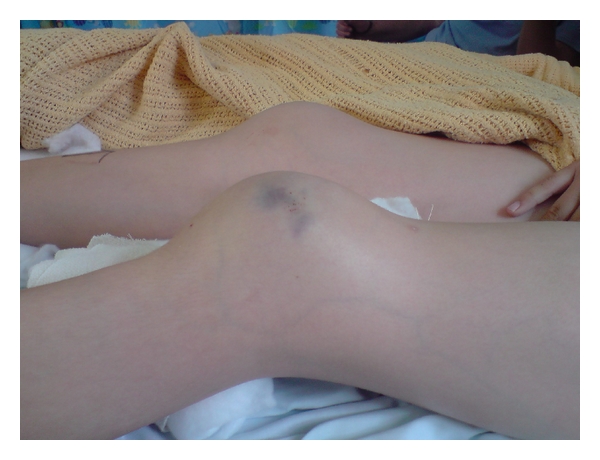
Clinical appearance of the knees on initial presentation.

**Figure 2:**
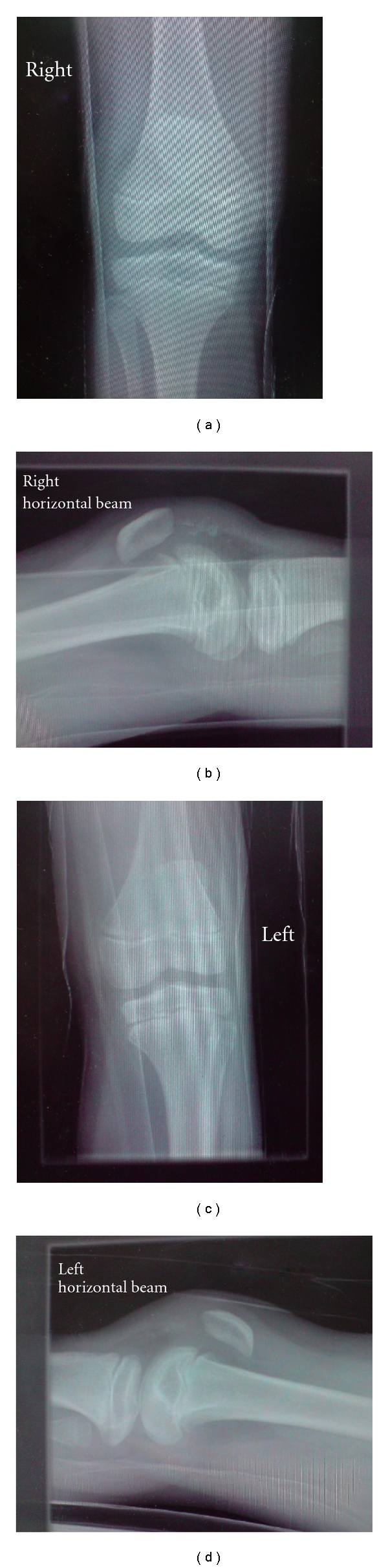
(a) AP radiograph of the right knee. (b) Lateral radiograph of the right knee. (c) AP radiograph of the left knee. (d) Lateral radiograph of the left knee.

**Figure 3:**
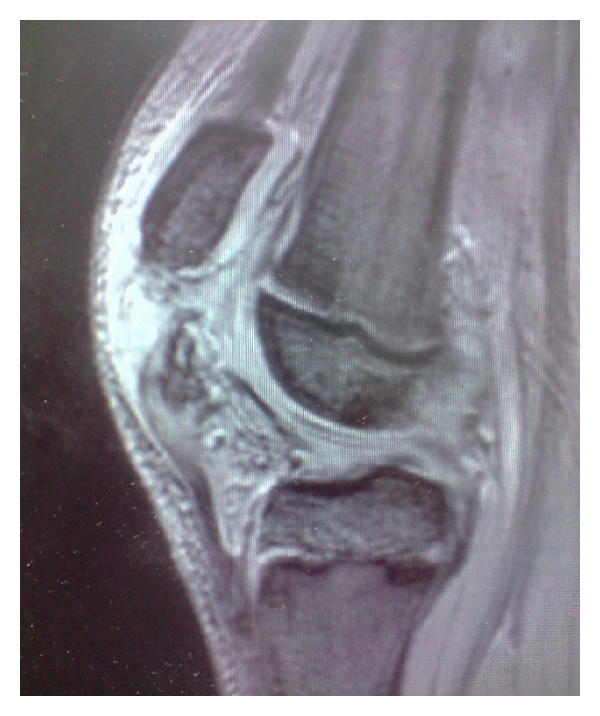
Sagittal T_2_ MRI of the right knee.

A healthy 11-year-old boy landed on his trampoline and complained immediately to his parents of bilateral knee pain. He was jumping vertically straight up and down at the time and no other person was on the trampoline. He had no significant past medical, drug, or family histories. He specifically had no history of anterior knee pain nor was he hypermobile (Beighton score 0). He attended hospital with bilateral knee pain with significant effusions, the left being larger clinically than the right (Figure 1). Radiographs of the knees were suspicious of sleeve fractures (Figures 2(a)–2(d)) and an MRI of both knees was organised. The MRI clearly revealed the extent of the displaced sleeve fractures (Figure 3).

**Figure 4:**
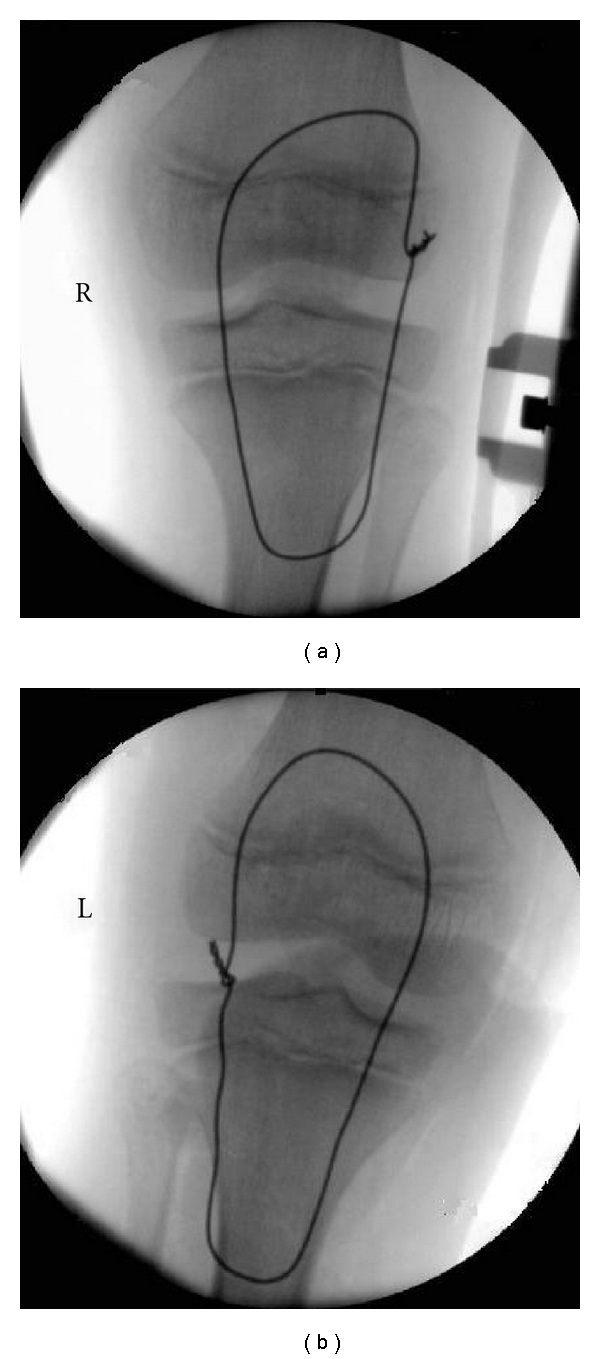
(a) Intra-operative image of the right knee. (b) Intra-operative image of the left knee.

He underwent open reduction and internal fixation of the injuries within 24 hours. A midline incision was used. The paratenon was incised in the midline and dissected off the proximal few centimetres of the proximal patellar tendon and the fracture site defined. The knee then underwent a copious lavage. Three transosseous tunnels were drilled in the coronal plane with a 2.5 mm drill. Number 5 Ethibond was then used to anatomically oppose the ends of the sleeve fracture. The construct was reinforced with a circlage wire, passing through the tibial tuberosity and above the superior pole of the patella, with the wire twisted so that it could be retrieved later through a small incision (Figures 4(a) and 4(b)). The torn extensor retinaculum was then repaired using absorbable sutures.

Postoperatively, the legs were immobilised in lightweight casting material in extension for a period of 6 weeks nonweightbearing. Followed by a hinged knee brace for 6 weeks, gradually increasing the range of motion and weight bearing status. Had the injury been unilateral then he would have been FWB in extension from surgery. The circlage wires were subsequently removed uneventfully at 6 months, prior to this he had regained full symmetrical range of motion (0–135 degrees) and was pain-free. He was allowed to return to full activity 6 weeks after removal of the circlage wires during which time he attended physiotherapy.

## 3. Discussion

Sleeve fractures are a type of paediatric avulsion fracture and were first described by Houghton and Ackroyd in 1979 [1]. A sleeve fracture is defined as an avulsion of a small bony fragment from the distal pole of the patella, along with its articular cartilage, periosteum, and retinaculum, which is pulled off from the main body of the structure [2]. Avulsion fractures can be classified too according to their location [3]. Patellar fractures in the skeletally immature are rare with an incidence of 1–6.5% of all fractures; of these only 5% occur at either pole as an avulsion fracture [2, 4, 5]. The greatest challenge of management is in the initial diagnosis. 

The literature demonstrates numerous problems due to delayed or misdiagnosis [2, 6–8]. Eliciting a salient history focusing on the mechanism of injury is important. Key features of a sleeve fracture are the absence of direct knee trauma. They are caused by an explosive eccentric contraction of the quadriceps muscle often seen in jumping activities, as in our case. The unusual nature of our case is that the mechanism led to bilateral “overloading” of the extensor mechanism. Physical examination may be difficult and you need to have a high index of suspicion from the mechanism. The examination may reveal decreased flexion, inability to extend (or an extensor lag) and a large effusion. It is of note that the knee may be capable of active extension if there is an intact posterior cartilaginous hinge in continuity [3, 5, 6]. Often the knee is very tender and swollen, just like our case; thus palpation of a high-riding patella may be all that is ascertained. A palpable gap may be felt if the fracture is significantly displaced with point tenderness [2, 4].

A lateral plain radiograph may help in making the diagnosis by demonstrating the avulsed bone fragment or flake and patella alta [1, 4, 9, 10]. If the avulsed fragment, consisting mainly of avulsed cartilage, has minimal bone within it then they can be easily missed [7, 8]. Other pathology seen on the AP or lateral radiographs are bipartite patella, accessory ossification centres or Sinding-Larsen-Johansson disease [11–13]. A sleeve fracture has been viewed by some as an advanced form of Sinding-Larsen-Johansson disease [5] although the treatment and management of this condition is by conservative means and is not an acute injury. Here, the lower pole of the patella, being inherently weak during growth, can undergo repetitive microtrauma during exercise, leading to partial avulsions which heal with calcification [13].

The use of MRI to assist in diagnosis and management of sleeve fractures, particularly in sagittal plane T_2_-weighted images, in the line of the patellar tendon, can help define the injury further. If the injury is obvious on plain films/clinically then some would consider MRI a luxury rather than a necessity. Studies describe how classical signal intensity pattern changes can help delineate further the extent of the cartilaginous injury and the relationship of the fracture fragments [9, 14]. This is due to the contrast between the high signal-intensity seen at a fracture line and the low signal-intensity of the cartilage. Bates concluded that an MRI should be performed as the outcome of conservative management of a nondisplaced injury is satisfactory, clearly the plain radiographs will not show you the information you require to make that decision [9]. An alternative imaging adjunct is the use of ultrasound. This may provide a rapid, safe, cost-effective method of imaging displacement of the sleeve fragment, especially if no displaced bony fragments are seen on the lateral radiograph [15].

Recommended management of displaced patella sleeve fractures is by open reduction and internal fixation to achieve a good functional result [1, 2, 7, 8, 16–19]. No study has shown superior results using different techniques due to small numbers involved. Treatment options include open reduction and internal fixation with transosseous nonabsorbable sutures [19], absorbable anchor sutures [17], and tension band wiring with sutures [1, 18, 20] and metal [7, 8, 21]. Careful repair of the torn extensor retinaculum is also advocated. If patient diagnosis and treatment is delayed or a displaced sleeve fracture is misdiagnosed and managed nonoperatively then outcomes will be unsatisfactory. Patients will tend to be pain-free but may exhibit extensor lag, prominence, and deformity of the patella and wasting of the quadriceps muscles [1, 2, 8, 18]. Patients with operative and non-operative management may have reduced knee flexion (from immobilisation in a cast) [22]. Problems associated specifically with operative management include ectopic bone formation [23] and transient ischaemic changes. Potentially, because the blood supply of the immature patella comes predominantly from the anterior surface of the distal pole, injury here or excessive surgical exposure may lead to avasular necrosis of the proximal pole [1, 2, 24].

This paper and literature review has highlighted a rare case of bilateral patellar sleeve fractures in a normal child. More importantly, the authors hope that this has refreshed minds with the often missed and misdiagnosed management of sleeve fractures.
